# Clinical and Structural Associations of Disability and Gait Performance in Patients With Rheumatoid Arthritis in Remission and Metatarsal Pain

**DOI:** 10.1002/jfa2.70133

**Published:** 2026-02-12

**Authors:** Rebeca Bueno Fermoso, Rosario Morales Lozano, Carmen Martínez Rincón, Pablo García Fernández, Juan Miguel López González, Maria Luz González Fernández

**Affiliations:** ^1^ Facultad de Enfermería, Fisioterapia y Podología Universidad Complutense de Madrid Madrid Spain; ^2^ Hospital Universitario de Villalba Madrid Spain

**Keywords:** disability, forefoot pain, gait velocity, joint damage, metatarsophalangeal dislocation, rheumatoid arthritis

## Abstract

**Background:**

Patients with rheumatoid arthritis (RA) may continue to experience foot‐related disability despite clinical remission. Foot involvement is associated with self‐reported disability and objective gait alterations. Foot involvement is heterogeneous; studying patients with localised forefoot pain may help clarify which structural and inflammatory factors are associated with functional impairment.

**Objectives:**

To identify clinical, structural and imaging factors associated with disability and gait performance in patients with RA in clinical remission with metatarsal‐region forefoot pain.

**Methods:**

Cross‐sectional study of 81 patients with RA in remission with metatarsal‐region forefoot pain. Outcomes were Foot Function Index disability and activity limitation (FFI‐D and FFI‐AL), gait velocity (GV) and double‐support time (DS). Associations were examined using Spearman correlations, age‐ and BMI‐adjusted individual linear regression, parsimonious multivariable linear regression and exploratory cluster analysis.

**Results:**

Mean (SD) FFI‐D and FFI‐AL were 29.8 (29.4) and 29.1 (27.7); gait velocity (GV) was 0.90 (0.34) m/s and double‐support time (DS) was 22.9 (8.2)% of the gait cycle. In bivariate and age/BMI‐adjusted analyses, graded structural measures showed more consistent associations with disability and gait outcomes than dichotomous/count variables. In parsimonious models, disability (FFI‐D/FFI‐AL) was independently associated with pain intensity (VAS) and first metatarsophalangeal joint stiffness/limited dorsiflexion (1stMTP), whereas gait performance was mainly associated with age/BMI, greater graded forefoot structural severity and 1stMTP. Cluster analysis identified a higher grey‐scale synovitis (GS)/lower structural‐burden group and a lower GS/higher structural‐burden group, with worse function and slower gait in the latter.

**Conclusions:**

In RA remission with metatarsal‐region forefoot pain, perceived disability is mainly associated with pain and forefoot stiffness, whereas gait performance is more closely related to age/BMI and graded forefoot structural severity. These findings support severity‐based region‐specific structural assessment alongside pain evaluation in clinical follow‐up, to avoid underestimating the independent contribution of structural damage to function and gait.

**Abbreviations**
BMI: Body mass index
CDAI: Clinical disease activity index
DAS28: Disease activity score in 28 joints
DIS: Number of MTP joints with clinical dislocation/subluxation (count; summed 0–10 per patient)
DIS‐grade: Summed radiographic grade of metatarsophalangeal joint dislocation (0–30)
DS: Double‐support time (percentage of gait cycle)
ER: Erosions (radiographic)
FFI: Foot Function Index
FFI‐AL: Foot Function Index—activity limitation subscale
FFI‐D: Foot Function Index—disability subscale
GS: Grey‐scale synovitis (ultrasound)
GV: Gait velocity
HAV: Hallux valgus
HAV‐grade: Manchester scale grade of hallux valgus (bilateral sum)
JSN: Joint space narrowing
1stMTP: First metatarsophalangeal joint
MTP: Metatarsophalangeal
PD: Power Doppler (ultrasound)
RA: Rheumatoid arthritis
ROM: Range of motion
SENS: Simple erosion narrowing score (radiographic)
SIS: Structural index score
SIS‐forefoot: Structural index score—forefoot component
SIS‐rearfoot: Structural index score—midfoot and rearfoot component
TB: Tailor's bunion (fifth MTP joint)
VAS: Visual analogue scale

## 1 Introduction

Rheumatoid arthritis (RA) affects the foot in up to 90% of patients over the disease course [1, 2], with the forefoot being the most frequently involved region [3, 4].

Normal joint motion of the foot is essential to allow forward progression during stance and gait, facilitating weight transfer, shock absorption and stability [5]. Both in early [5] and advanced stages of RA [6, 7], involvement of the feet and lower limbs compromises daily‐life activities, particularly ambulation and weight‐bearing [8], thereby contributing substantially to disability [9, 10] and mobility limitation. To assess this disability, both self‐reported measures [11] and objective performance‐based tests are used, although these do not always correlate [12, 13, 14].

Although current therapeutic strategies have improved inflammatory control and increased remission rates in RA, clinical remission mainly reflects low systemic disease activity and may not fully capture local structural and biomechanical impairment at the foot level. In this context, functional disability, pain and structural joint damage may represent constructs that are related to, but not equivalent to, inflammatory disease activity, especially in patients who meet clinical remission criteria [15, 16]. Foot involvement in RA has been associated with functional disability, activity limitation and gait impairment, and these aspects have been addressed in recent studies focussing on foot function and gait‐related outcomes in patients with rheumatoid arthritis [17, 18, 19].

Foot involvement in RA is not homogeneous and may follow different regional patterns (forefoot, midfoot and hindfoot) [20, 21]. Such regional heterogeneity underscores the importance of region‐specific assessment when evaluating functional outcomes as different patterns of foot involvement may differentially affect global gait parameters [21].

Previous studies often include heterogeneous populations with varying levels of inflammatory activity and mixed patterns of foot involvement, which may mask associations specific to each clinical pattern [21, 22, 23]. Moreover, some global forefoot scores rely on dichotomous or count‐based items and be less sensitive to the severity and mechanical relevance of structural involvement as they do not incorporate graded metrics [22]. Additionally, certain studies have not considered clinically relevant factors, such as age [14, 24], which also influence physical function.

In this context, the present study aimed to identify clinical, structural and imaging factors associated with self‐reported disability and gait performance, assessed using the subscales of the Foot Function Index (FFI), gait velocity (GV) and double‐support time (DS), in patients with RA in clinical remission and metatarsal‐region forefoot pain. As an exploratory aim, clinical–structural phenotypes were characterised through cluster analysis (hypothesis‐generating).

## 2 Materials and Methods

### 2.1 Study Design and Ethics

Cross‐sectional observational study, including patients with RA, diagnosed by a rheumatologist in routine clinical practice, in accordance with the 2010 ACR/EULAR classification criteria [25]. In clinical remission, defined as Disease Activity Score in 28 joints (DAS28) [26] < 2.6 and Clinical Disease Activity Index (CDAI) ≤ 2.8. In accordance with the Declaration of Helsinki and Good Clinical Practice guidelines. Approved by the Ethics Committee of Hospital Clínico San Carlos (Madrid) (Ref. 21/719‐E). Written informed consent was obtained from all participants prior to inclusion.

### 2.2 Setting and Recruitment

All evaluations were conducted at the Rheumatic Foot Unit of the University Podiatry Clinic, School of Nursing, Physiotherapy and Podiatry, Universidad Complutense de Madrid (UCM). Patients were referred from five public hospitals in the Community of Madrid. Consecutive recruitment was carried out between January 2022 and December 2024 by podiatrists with more than 15 years of clinical experience.

### 2.3 Examiners and Blinding

A first examiner reviewed the medical records, performed patient screening, conducted the clinical interview and anonymised the radiographs. A second examiner confirmed the location of pain and performed the clinical examination and gait assessment. A third examiner, blinded to the clinical findings, performed and scored the ultrasound assessment. A fourth examiner, blinded to both clinical and ultrasound findings, scored the previously anonymised radiographs using a predefined reading list.

For quality control, intra and inter‐rater reliability was assessed in a subsample selected and coded by the second examiner (15 ultrasound and 15 radiographic examinations and 75 MTP joints each). This subsample was subsequently sent to the third and fourth examiners (each with more than 30 years of clinical experience and expertise in rheumatic patients and musculoskeletal imaging) for reevaluation to estimate intra and inter‐rater reliability. For inter‐rater reliability, both imaging examiners independently scored the same stored images; for intrarater reliability, each examiner repeated the scoring at a second time point. Agreement was high (coefficients > 0.80).

### 2.4 Inclusion and Exclusion Criteria

Inclusion criteria were as follows: confirmed RA diagnosis by a rheumatologist; clinical remission (DAS28 < 2.6 and CDAI ≤ 2.8) [26]; presence of metatarsal‐region forefoot pain; signed informed consent and availability of weight‐bearing dorsoplantar foot radiographs obtained within the previous six weeks.

Forefoot pain was defined as patient‐reported pain predominantly located at the metatarsal region, confirmed during clinical interview and localised by the patient to the forefoot area (metatarsal heads/MTP region). Pain intensity was recorded using a 0–10 VAS referring specifically to forefoot pain.

Exclusion criteria were as follows: other rheumatic or neuromuscular diseases; diabetes mellitus; foot ulcers or tumours; recent lower‐limb trauma within the previous 6 months; history of foot/ankle surgery; use of foot orthoses or corticosteroid injections within the previous 3 months; cognitive impairment; inability to complete the assessment and incomplete data or missing information in any primary study variable.

### 2.5 Sample Size and Bias Control

Sample size was estimated a priori using G*Power (linear multiple regression, fixed model and *R* ^2^ deviation from zero). Assuming an expected *R* ^2^ = 0.20 (Cohen's *f* ^2^ = 0.25), *α* = 0.05, 80% power and up to 8 predictors in the largest planned model, the minimum required sample size was 69 participants. A total of 81 participants were ultimately included. The assumed *R* ^2^ was used as a conservative planning value (moderate effect size) for multifactorial clinical models and was not derived from the observed data.

Several strategies were implemented to reduce bias as follows: consecutive recruitment; clinical examination and gait assessment performed by the same experienced examiner; ultrasound and radiographic evaluations conducted by independent examiners blinded to clinical findings and questionnaires administered through clinical interview to minimise comprehension bias.

### 2.6 Variables and Procedures

General variables were as follows: age, weight, height, BMI, RA duration and current medication (clinical interview and review of medical history).

Perceived disability was assessed using the disability subscale of the Foot Function Index (FFI‐D) [27], and mobility limitation using the corresponding activity limitation subscale (FFI‐AL), both in their validated Spanish versions. The FFI pain subscale was not included because pain intensity was assessed separately using the visual analogue scale (VAS) and including both measures could introduce collinearity.

Pain was assessed using VAS [28], referring specifically to forefoot pain (metatarsal region) experienced by the patient.

Clinical deformities were assessed using the items of the structural index score (SIS) [22], distinguishing between forefoot (SIS‐forefoot) and midfoot–rearfoot (SIS‐rearfoot). Both feet were evaluated separately, and a total patient score was obtained. The SIS‐forefoot is the sum of as follows: presence of hallux valgus (HAV) (0–1), number of metatarsophalangeal (MTP) joints with dislocation (DIS; 0–5), presence of fifth MTP joint exostosis (0–1) and presence of claw or hammer toes (digital deformities) (0–5) [22, 29]. The SIS‐rearfoot includes calcaneal valgus/varus (angle: 0°–5° = 0; 6°–10° = 1; 11°–15° = 2 and > 15° = 3), ankle range of motion (ROM) (46°–60° = 0; 31°–45° = 1 and 15°–30° = 2; < 15° = 3) and pes planus/cavus (absent = 0 and present = 1), assessed with a calibrated goniometer.

HAV severity was assessed using the Manchester scale [30, 31] (HAV‐grade), scoring 0–3 per foot and 0–6 per patient. First MTP joint dorsiflexion (stiffness/limitation) (1stMTP) was assessed in nonweight‐bearing conditions [32]. The examiner stabilised the first metatarsal whilst applying passive dorsiflexion to the hallux until firm end‐range. A small goniometer was placed on the medial aspect of the 1stMTP joint, with the fulcrum centred over the joint line; the stationary arm was aligned with the longitudinal axis of the first metatarsal and the moving arm with the longitudinal axis of the proximal phalanx of the hallux. For scoring purposes, dorsiflexion was categorised as follows: no limitation (≥ 40°), hallux limitus (10°–39°) and hallux rigidus (< 10°), yielding a score of 0–2 per foot and 0–4 per patient [33]. Three measurements per foot were taken and averaged. Presence of tailor’s bunion (TB) at the fifth MTP joint was also recorded [34].

Ultrasound was performed with the patient seated, using a high‐frequency linear transducer (LA3‐14AD) 10–14 MHz and a Samsung HS50 system. Grey‐scale (GS) synovitis was assessed in MTP joints 1–5 (MTP1–5), and power Doppler (PD) signal was evaluated using the OMERACT scale [35, 36]. For statistical purposes, the number of joints with synovitis (0–5 per foot and 0–10 per patient) was recorded.

Weight‐bearing dorsoplantar radiographs of the forefoot were evaluated using the Simple Erosion Narrowing Score (SENS) [37], recording erosions (ER) and joint space narrowing (JSN) in the MTP joints and the hallux interphalangeal joint (total ER and JSN: 0–6 per foot and 0–12 per patient; total SENS: 0–12 per foot and 0–24 per patient).

MTP dislocation (DIS‐grade) was assessed using the radiographic method described by Ohashi et al. [38]. This represents a static radiographic grading of MTP joint dislocation. Grading was based on weight‐bearing dorsoplantar radiographs. 0 = absent; 1 = < 50% dislocation; 2 = > 50% dislocation and 3 = complete dislocation. Scores ranged from 0 to 15 per foot and 0–30 per patient.

Gait analysis was conducted in a dedicated laboratory using a Footscan plantar pressure measurement system (RSscan International, Olen, Belgium) with a 2 m plate (4 sensors/cm^2^) and a 3D‐Box interface. Data were sampled at 500 Hz and processed using Scientific Footscan software. Participants walked barefoot at their self‐selected speed along a walkway that allowed them to start walking before reaching the plate (allowing at least three preceding steps) and to continue walking afterwards, so that foot contact occurred during continuous gait. Prior to data collection, a brief familiarisation period (1 min, adjustable according to tolerance) was performed to ensure comfortable walking. A minimum of three trials per participant were recorded, and mean values were used for the analysis of spatiotemporal variables (walking speed and double support). The system was calibrated prior to each session.

### 2.7 Statistical Analysis

Continuous variables are reported as mean ± SD and categorical variables as *n* (%). Primary outcomes were FFI‐D and FFI‐AL (0–100; higher scores = worse function), gait velocity (GV, m/s) and double‐support time (DS, % gait cycle). Spearman correlations (*ρ*, two‐tailed) were used to describe bivariate associations and screen for redundancy/collinearity among candidate predictors.

To inform model specification, we fitted age‐ and BMI‐adjusted linear regression models for each outcome by adding each candidate predictor individually to a base model (age +BMI). For each model, we report *B*, SE, standardised *β*, *p*‐values and Δ*R* ^2^ (F‐change *p*‐value); full results are provided in the (Supporting Information S1: Tables S2 and S3).

For each outcome, we then built predefined parsimonious multivariable linear regression models to estimate independent associations whilst limiting collinearity. Predictors were selected a priori to represent key domains (pain, local inflammation and structural/mechanical severity) and to maximise clinical interpretability whilst minimising collinearity. Age and BMI were forced in all models, together with VAS and GS synovitis. Structural involvement was represented by SIS‐rearfoot and two graded forefoot measures capturing complementary constructs (DIS‐grade and 1stMTP). Alternative specifications replacing correlated structural measures (e.g., SIS‐forefoot or radiographic scores) were assessed in sensitivity analyses (Supporting Information S1: Tables S4–S7). Model assumptions were checked using residual diagnostics; collinearity was assessed using VIF. Because age and RA duration were strongly correlated, age was retained as the primary covariate and RA duration was evaluated descriptively and in sensitivity analyses. Statistical significance was set at *α* = 0.05 (two‐tailed).

An exploratory cluster analysis was performed using z‐standardised GS, DIS‐grade, HAV‐grade and 1stMTP. Hierarchical clustering (Ward's method and squared Euclidean distance) was used, and the number of clusters was selected by visual inspection of the dendrogram; the selected solution was then consolidated using k‐means clustering. Clusters were compared for age/RA duration and outcomes using Student's t test or Mann–Whitney *U* test as appropriate. Analyses were conducted in SPSS v29.

## 3 Results

Eighty one patients with RA in remission and metatarsal‐forefoot pain, of whom 95.1% (77) were women. The mean age was 63.2 ± 11.5 years (36–85), and the mean RA duration was 21.2 ± 15.0 years (3–61).

Medication use was as follows: 48.1% glucocorticoids, 71.6% conventional synthetic disease‐modifying antirheumatic drugs and 45.7% biologic agents. 48.1% nonsteroidal anti‐inflammatory drugs and 22.2% paracetamol. 9.9% antiplatelet agents, 30.9% antihypertensive drugs, 38.3% lipid‐lowering therapy and and 19.8% thyroid hormone replacement.

Descriptive characteristics of the cohort are summarised in Table 1.

**TABLE 1:** Descriptive characteristics of the study sample (*n* = 81).

Variable	Mean (SD)	Range (min–max)
Pain VAS (0–10)	7.56 (2.00)	1–10
BMI (kg/m^2^)	25.73 (4.05)	18.22–35.16
RA duration (years)	21.21 (14.95)	3–61
FFI‐D (0–100)	29.80 (29.39)	0–100
FFI‐AL (0–100)	29.06 (27.69)	0–100
GV (m/s)	0.90 (0.34)	0.23–1.57
DS (% gait cycle)	22.92 (8.23)	14.61–50.86
GS (0–10 joints)	4.72 (3.51)	0–10
PD (0–10 joints)	0.15 (0.59)	0–3
SENS (0–24)	13.26 (6.74)	0–24
JSN (0–12)	7.83 (4.23)	0–12
ER (0–12)	5.16 (3.59)	0–12
SIS‐forefoot (0–24)	14.27 (7.00)	0–22
SIS‐rearfoot (0–14)	5.08 (2.36)	0–10
Digital deformities (0–10)	6.17 (3.00)	0–8
DIS (0–10)	5.06 (3.83)	0–10
DIS‐grade (0–30)	12.59 (10.05)	0–30
HAV (0–2)	1.30 (0.94)	0–2
HAV‐grade (0–6)	2.40 (2.20)	0–6
1stMTP (0–4)	1.78 (1.30)	0–4
TB (0–2)	1.21 (0.98)	0–2

Abbreviations: 1stMTP, first metatarsophalangeal joint stiffness (bilateral sum); BMI, body mass index; digital deformities, number of toes with claw or hammer deformity; DIS, number of metatarsophalangeal (MTP) joints with dislocation; DIS‐grade, summed dislocation grade in MTP joints (0–30); DS, double‐support time; ER, erosions (radiographic); FFI‐AL, Foot Function Index—activity limitation subscale; FFI‐D, Foot Function Index—disability subscale; GS, grey‐scale synovitis (number of MTP joints with synovitis); GV, gait velocity; HAV, presence of hallux valgus; HAV‐grade, Manchester scale hallux valgus grade (bilateral sum); JSN, joint space narrowing (radiographic); PD, power doppler signal (number of MTP joints with PD); RA duration, rheumatoid arthritis duration in years; SENS, simple erosion narrowing score (total radiographic score); SIS‐forefoot, structural index score—forefoot component; SIS‐rearfoot, structural index score—midfoot and rearfoot component; TB, tailor’s bunion (5th MTP joint); VAS, visual analogue scale.

### 3.1 Bivariate Analyses

In bivariate analyses (Table 2), graded forefoot measures (DIS‐grade, HAV‐grade and 1stMTP) showed the most consistent correlations with worse FFI scores and lower GV. SIS‐rearfoot showed moderate correlations with disability, whereas SIS‐forefoot correlated mainly with GV. GS synovitis correlated inversely with FFI scores and positively with GV, whereas PD showed only weak associations with pain (VAS). Radiographic/structural indices were strongly intercorrelated (Supporting Information S1: Table S1).

**TABLE 2:** Spearman's correlation coefficients (*ρ*) between clinical, structural, inflammatory and functional variables in patients with rheumatoid arthritis and metatarsal‐region forefoot pain.

Variable	FFI‐D *ρ* (*p*)	FFI‐AL *ρ* (*p*)	GV *ρ* (*p*)	DS *ρ* (*p*)	VAS *ρ* (*p*)
SIS‐forefoot	0.15 (0.18)	0.15 (0.19)	−0.39 (< 0.001)	0.18 (0.12)	−0.04 (0.72)
SIS‐rearfoot	0.42 (< 0.001)	0.41 (< 0.001)	−0.22 (0.05)	0.20 (0.08)	0.30 (0.01)
DIS‐grade	0.22 (0.05)	0.22 (0.05)	−0.49 (< 0.001)	0.19 (0.10)	0.05 (0.67)
HAV‐grade	0.29 (0.01)	0.29 (0.01)	−0.42 (< 0.001)	0.26 (0.02)	0.05 (0.68)
TB	0.14 (0.23)	0.13 (0.25)	−0.04 (0.72)	0.26 (0.03)	0.18 (0.11)
1stMTP	0.44 (< 0.001)	0.44 (< 0.001)	−0.54 (< 0.001)	0.35 (0.002)	0.23 (0.04)
SENS	0.20 (0.07)	0.19 (0.09)	−0.53 (< 0.001)	0.21 (0.07)	0.15 (0.17)
GS	−0.34 (0.002)	−0.33 (0.002)	0.39 (< 0.001)	−0.22 (0.06)	−0.15 (0.19)
PD	0.04 (0.75)	0.04 (0.71)	−0.08 (0.50)	−0.16 (0.16)	0.23 (0.04)
VAS	0.39 (< 0.001)	0.39 (< 0.001)	−0.23 (0.04)	0.10 (0.40)	—
BMI	0.08 (0.47)	0.07 (0.53)	−0.09 (0.43)	0.29 (0.01)	−0.02 (0.88)
RA duration	0.22 (0.05)	0.22 (0.05)	−0.35 (0.001)	0.23 (0.05)	0.20 (0.07)
Age	0.20 (0.08)	0.20 (0.08)	−0.55 (< 0.001)	0.20 (0.08)	0.03 (0.77)

*Note: ρ*, Spearman's correlation coefficient; *p*, two‐tailed value.

Abbreviations: 1stMTP, first metatarsophalangeal stiffness/limitation score; Age, age (years); BMI, body mass index; DIS‐grade, summed metatarsophalangeal dislocation grade (0–30); DS, double‐support time; FFI‐AL, Foot Function Index–activity limitation subscale; FFI‐D, Foot Function Index–disability subscale; GS, grey‐scale synovitis; GV, gait velocity; HAV‐grade, hallux valgus grade (Manchester scale and bilateral sum); PD, power doppler; RA duration, rheumatoid arthritis duration (years); SENS, simple erosion narrowing score; SIS‐forefoot, structural index score–forefoot component; SIS‐rearfoot, structural index score–midfoot and rearfoot component; TB, tailor’s bunion; VAS, visual analogue scale.

### 3.2 Multivariable Analysis

Age‐ and BMI‐adjusted single‐predictor linear regression models (each candidate predictor entered separately) are reported in the (Supporting Information S1: Tables S2 and S3).

In the base model, age was associated with higher disability (FFI‐D/FFI‐AL) and lower GV; BMI showed a borderline association with DS, with no relevant associations for the remaining outcomes.

Forefoot structural domain. Overall, graded forefoot severity metrics (1stMTP, DIS‐grade and HAV‐grade) showed the most consistent associations with disability and/or gait parameters in age‐ and BMI‐adjusted models. In contrast, lower‐resolution presence/count‐based measures (e.g., DIS, HAV, digital deformities and SIS‐forefoot) tended to show weaker or less consistent associations. Within composite clinical indices, SIS‐rearfoot showed more consistent associations with disability than SIS‐forefoot, whereas associations with gait parameters were more modest.

Inflammatory domain. GS (number of MTP joints with synovitis) was inversely associated with FFI‐D/FFI‐AL and positively associated with GV; the association with DS was weaker and not statistically significant. Power Doppler (PD) showed no significant associations with outcomes.

Pain. Pain intensity (VAS) was consistently associated with higher FFI‐D and FFI‐AL scores, whereas associations with gait parameters were weaker and less consistent.

### 3.3 Parsimonious Multivariable Models

Because several structural indices were intercorrelated (Supporting Information S1: Table S1), parsimonious multivariable models are presented in Table 3. Sensitivity analyses are provided in the (Supporting Information S1: Tables S4–S7).

**TABLE 3:** Parsimonious multivariable models.

Outcome (*R* ^2^; Adj *R* ^2^)	Predictor	*B*	SE	*β*	95% CI	*p*
FFI‐D (0.435; 0.380)	Age (years)	0.00	0.30	0.000	−0.60 to 0.60	0.997
	BMI (kg/m^2^)	0.13	0.67	0.018	−1.20 to 1.47	0.843
	VAS	3.80	1.40	0.258	1.02 to 6.58	0.008
	GS	−1.52	0.87	−0.181	−3.25 to 0.22	0.086
	SIS‐rearfoot	1.28	1.32	0.103	−1.35 to 3.92	0.334
	DIS‐grade	0.47	0.34	0.161	−0.20 to 1.14	0.165
	1stMTP	6.98	2.58	0.310	1.85 to 12.12	0.008
FFI‐AL (0.389; 0.330)	Age (years)	0.09	0.29	0.037	−0.50 to 0.67	0.761
	BMI (kg/m^2^)	0.00	0.66	0.000	−1.31 to 1.31	0.998
	VAS	3.66	1.37	0.264	0.94 to 6.38	0.009
	GS	−1.14	0.85	−0.144	−2.84 to 0.56	0.186
	SIS‐rearfoot	0.89	1.29	0.076	−1.69 to 3.47	0.492
	DIS‐grade	0.33	0.33	0.120	−0.33 to 0.98	0.320
	1stMTP	6.60	2.52	0.311	1.57 to 11.63	0.011
GV (0.498; 0.450)	Age (years)	−0.007	0.003	−0.219	−0.013 to 0.000	0.051
	BMI (kg/m^2^)	−0.007	0.007	−0.084	−0.022 to 0.008	0.338
	VAS	−0.008	0.015	−0.047	−0.039 to 0.022	0.601
	GS	0.016	0.010	0.162	−0.003 to 0.035	0.102
	SIS‐rearfoot	0.015	0.015	0.106	−0.014 to 0.044	0.292
	DIS‐grade	−0.011	0.004	−0.321	−0.018 to −0.004	0.004
	1stMTP	−0.074	0.028	−0.283	−0.131 to −0.018	0.011
DS (% gait cycle) (0.273; 0.200)	Age (years)	−0.08	0.11	−0.106	−0.29 to 0.13	0.454
	BMI (kg/m^2^)	0.57	0.22	0.282	0.14 to 1.00	0.010
	VAS	0.92	0.46	0.222	0.00 to 1.84	0.051
	GS	−0.02	0.28	−0.010	−0.59 to 0.54	0.933
	SIS‐rearfoot	−0.41	0.43	−0.118	−1.26 to 0.45	0.349
	DIS‐grade	0.18	0.11	0.216	−0.05 to 0.40	0.119
	1stMTP	2.13	0.84	0.343	0.46 to 3.80	0.013

*Note:* All models include age, BMI, VAS, GS, SIS‐rearfoot, DIS‐grade and 1stMTP. FFI‐D and FFI‐AL range from 0 to 100 (higher scores = worse function); GV is in m/s and DS is % of the gait cycle. *B*, unstandardised regression coefficient (per 1‐unit increase in predictor).

Abbreviations: 1stMTP, first metatarsophalangeal stiffness/limitation score;*β*, standardised coefficient. Age, age (years); BMI, body mass index (years); BMI (kg/m^2^); CI, 95% confidence interval; DIS‐grade, summed MTP dislocation grade; DS, double‐support time (% of the gait cycle); FFI‐AL, Foot Function Index—activity limitation; FFI‐D, Foot Function Index—disability; GS, grey‐scale synovitis; GV, gait velocity (m/s); SE, standard error; SIS‐rearfoot, structural index score—rearfoot component; VAS, visual analogue scale.

In the parsimonious multivariable models (Table 3), disability outcomes (FFI‐D and FFI‐AL) were independently associated with pain intensity (VAS) and 1stMTP. Gait velocity was independently associated with DIS‐grade and 1stMTP. Double‐support time was independently associated with BMI and 1stMTP. Age showed a borderline association with GV (*p* = 0.051), and VAS showed a borderline association with DS (*p* = 0.051).

For clinical interpretability, in the parsimonious models (Table 3), a 1‐point increase in VAS (0–10) was associated with +3.80 points in FFI‐D and +3.66 points in FFI‐AL; a one‐point increase in 1stMTP score was associated with +6.98 (FFI‐D), +6.60 (FFI‐AL), −0.074 m/s (GV) and +2.13% points in DS (% of the gait cycle) and a one‐point increase in DIS‐grade was associated with −0.011 m/s in GV.

### 3.4 Cluster‐Derived Phenotypes

Exploratory (hypothesis‐generating) cluster analysis supported a two‐cluster solution (Table 4). Cluster 1 (Inflammatory; *n* = 57) presented higher GS and lower deformity/stiffness (lower DIS‐grade, HAV‐grade and 1stMTP), whereas Cluster 2 (Structural/mechanical; *n* = 24) showed lower GS and greater deformity/stiffness (higher DIS‐grade, HAV‐grade and 1stMTP). Clusters differed in age and RA duration (both *p* < 0.001) and in functional outcomes: Cluster 2 showed higher FFI‐D and FFI‐AL and lower GV (*p* < 0.001), whereas DS did not reach statistical significance (*p* = 0.070).

**TABLE 4:** Cluster‐derived phenotypes.

Variable	Cluster 1 inflammatory (*n* = 57)	Cluster 2 structural/mechanical (*n* = 24)	*p* value
Age (years)	58.04 ± 8.90	75.38 ± 6.74	< 0.001
RA duration (years)	16.86 ± 12.21	31.54 ± 16.01	< 0.001
VAS (0–10)	7.39 ± 2.08	7.96 ± 1.78	0.242
GS (0–10)	5.65 ± 3.23	2.50 ± 3.19	< 0.001
DIS‐grade (0–30)	7.65 ± 6.39	24.33 ± 6.92	< 0.001
HAV‐grade (0–6)	1.58 ± 1.55	4.33 ± 2.33	< 0.001
1stMTP (0–4)	1.33 ± 1.15	2.83 ± 1.01	< 0.001
FFI‐AL (0–100)	21.65 ± 18.19	46.67 ± 37.44	< 0.001
FFI‐D (0–100)	21.30 ± 17.68	50.00 ± 40.49	< 0.001
GV (m/s)	1.05 ± 0.27	0.56 ± 0.25	< 0.001
DS (% gait cycle)	21.90 ± 7.12	25.78 ± 10.47	0.070

Abbreviations: 1stMTP stiffness, first metatarsophalangeal joint stiffness score; DIS‐grade, summed metatarsophalangeal dislocation grade (0–30); DS, double‐support time; FFI‐AL, Foot Function Index—activity limitation subscale; FFI‐D, Foot Function Index—disability subscale; GS, grey‐scale synovitis (number of MTP joints with synovitis); GV, gait velocity; HAV‐grade, hallux valgus grade (Manchester scale, bilateral sum); RA duration, rheumatoid arthritis duration in years; VAS, visual analogue scale.

## 4 Discussion

This study addresses a common limitation in the literature when evaluating specific patterns of foot involvement or pain: the inclusion of heterogeneous samples (forefoot, midfoot and hindfoot involvement; varying levels of inflammatory activity and nongraded scales), which can dilute forefoot‐specific associations.

By restricting the cohort to patients with RA in clinical remission and metatarsal pain and by using graded metrics (1stMTP, DIS‐grade and HAV‐grade) together with radiographic JSN, we observed that disability (FFI‐D and FFI‐AL) and gait performance (GV and DS) were more strongly associated with graded forefoot structural measures than with ultrasound inflammatory markers, whereas pain intensity (VAS) primarily influenced self‐reported outcomes and, to a lesser extent, objective gait parameters.

Overall, forefoot damage and deformity were associated with all four outcomes, with effect sizes depending on the specific scale or item evaluated. Pain intensity was mainly related to FFI‐D/FFI‐AL and, more modestly, to DS, whereas higher GS tended to occur in participants with lower structural burden and better functional profiles (lower disability and slightly higher GV). Cluster analysis identified two phenotypes: an inflammatory phenotype (higher GS and lower deformity) and a structural/mechanical phenotype (lower GS and higher stiffness/dislocation/HAV), the latter showing worse FFI scores and lower gait velocity, consistent with the multivariable models. This likely reflects differences in age/disease duration and structural burden rather than a beneficial effect of synovitis.

Regarding structural evaluation (deformities and joint damage), graded forefoot metrics outperformed simple counts. The 1stMTP, DIS‐grade and HAV‐grade were more consistently associated with FFI‐D/FFI‐AL and GV than composite indices (e.g., SIS‐forefoot) or simple counts/binary measures (HAV, DIS and digital deformities). Our findings refine those reported by Gaino et al. [22]. In their more heterogeneous sample, with mixed foot involvement patterns, the composite forefoot index (SIS‐forefoot) was not clearly associated with disability, whereas certain single clinical variables, such as hallux valgus or MTP subluxations, showed functional relevance. In our more homogeneous cohort, patients with RA in remission and metatarsal pain, neither the SIS‐forefoot nor simple variables (HAV, DIS and digital deformities) consistently discriminated disability or gait performance, likely because the mere presence of forefoot involvement is almost universal and offers limited discriminatory power. In this context, graded metrics (1stMTP, DIS‐grade and HAV‐grade) best captured variability in function and gait, reinforcing that what we measure and how we grade it is as important as the region assessed. Among the structural variables evaluated, limitation of the 1stMTP emerged as one of the most consistent predictors in line with Bal et al. [14]. This item is not included in the SIS‐forefoot, which supports the lack of association of this index reported in different studies [22, 29]. This pattern was also reflected in the cluster analysis, where the structural/mechanical phenotype (higher stiffness/dislocation/HAV) showed worse disability, greater activity limitation, lower GV and a trend towards higher DS.

The literature suggests a substantial functional contribution of hindfoot and midfoot involvement [20, 21, 22, 23, 29, 39]. In our forefoot‐focused phenotype, rearfoot‐related clinical measures (SIS‐rearfoot) showed associations with disability outcomes, although comparisons with forefoot metrics should be interpreted cautiously given the different assessment approaches (clinical composite vs. graded and partly radiographic forefoot measures). Nonetheless, the hindfoot retained a measurable functional influence, suggesting that it should be systematically assessed even when pain is localised to the forefoot.

Radiographically, JSN showed more consistent associations with the outcomes than erosions in line with studies linking joint space narrowing to greater disability [40, 41]. In addition, the impact on GV was more evident for radiographic scores than for simple clinical counts, suggesting that the cumulative number of structurally affected joints penalises gait efficiency. Discrepancies with studies reporting no such relationship may be related to instrumental heterogeneity (e.g., scales focused on erosions or global outcomes such as the HAQ) and differences in populations or activity levels [7]. In any case, the influence of these characteristics on gait reinforces the need to include specific foot assessment, which is often underestimated in clinical practice [42].

Pain and disease activity are generally considered variables strongly related to disability [7]. Foot pain has also been associated with gait alterations such as reduced GV [43] and increased DS. In our cohort, VAS pain was associated with all outcomes in bivariate analyses, but after adjusting for age and BMI it remained primarily associated with self‐reported disability (FFI‐D/FFI‐AL). This pattern suggests that self‐report is more influenced by pain intensity, whereas objective performance depends more on structural and mechanical determinants. DS showed a more modest association with VAS than with forefoot clinical variables and BMI, reinforcing that the effect of pain on temporal gait parameters is secondary to mechanical factors.

When compared with the literature, our gait parameters were similar to those described in older adults with foot pain by Mickle et al. [43] who reported GV around 0.93–0.94 m/s in painful versus around 1.03 m/s in nonpainful individuals and DS 25.9%–26.5%, versus 0.90 m/s and 22.9% in our cohort and lower than those described in healthy subjects by Turner et al. [39] (around 1.20 m/s). In our adjusted models, pain intensity was not the main factor associated with gait performance; instead, structural forefoot variables (1stMTP stiffness, DIS‐grade and SENS/JSN) and age accounted for most of the variability, suggesting an additional mechanical burden beyond pain.

In this regard, Laroche et al. [44] observed that limited MTP ROM was associated with reduced GV and stride length at natural pace, supporting the mechanical plausibility of our findings. Similarly, van der Leeden et al. [8] reported more pronounced gait alterations in association with structural damage, highlighting the functional relevance of structural factors.

## 5 Conclusions

In patients with RA in clinical remission and metatarsal‐region forefoot pain, perceived disability (FFI‐D/FFI‐AL) was independently associated with pain intensity and 1stMTP stiffness/limitation, whereas gait performance was more strongly associated with age/BMI and graded forefoot structural severity (DIS‐grade) and 1stMTP stiffness. Grey‐scale synovitis clustered with a younger, lower structural‐burden phenotype and should be interpreted as a phenotype/stage marker rather than a protective factor. Overall, graded forefoot metrics showed more consistent associations with functional and gait outcomes than dichotomous/count measures, supporting severity‐based region‐specific structural assessment together with explicit pain grading in clinical follow‐up.

### 5.1 Clinical Implications

Graded forefoot structural metrics capture clinically relevant severity; dichotomous/count measures may miss meaningful information and underestimate associations with function and gait. Across age‐ and BMI‐adjusted models and sensitivity analyses, forefoot stiffness/limited first MTP dorsiflexion showed the most consistent associations with both self‐reported disability and gait parameters, whereas graded/radiographic structural severity (e.g., DIS‐grade and JSN/SENS, depending on model specification) was more strongly related to gait performance. Pain severity was independently associated with perceived disability; therefore, optimising pain management remains essential even in clinical remission. Together, the differential contributions of structural severity to objective gait performance and pain to patient‐reported disability support considering both constructs in assessment and follow‐up as they provide complementary information on functional impact. A whole‐foot approach is advisable; rearfoot findings may provide complementary clinical context, although comparisons with graded forefoot measures should be interpreted cautiously given the composite clinical nature of the SIS‐rearfoot.

### 5.2 Limitations and Strengths

Several limitations should be considered. The cross‐sectional design precludes temporal inference and the findings should be interpreted as associations within this clinical phenotype. Several radiographic indices were intercorrelated as expected for overlapping constructs of structural damage; to address redundancy and improve interpretability, we used parsimonious multivariable specifications. Given the number of tests performed across multiple outcomes and candidate predictors, the risk of type I error cannot be excluded; therefore, borderline associations should be interpreted cautiously. The cohort comprised mainly women with RA in clinical remission and metatarsal‐region forefoot pain recruited from a specialised clinic, and patients with prior foot/ankle surgery were excluded; generalisability to men, patients with active disease, different pain phenotypes, nonspecialised settings or postsurgical populations may therefore be limited. Because the outcomes reflect global locomotor performance and no non‐RA comparator group with similar metatarsal pain was included, the observed patterns cannot be attributed specifically to RA versus noninflammatory/mechanical causes of forefoot pain. Plate‐based barefoot assessment may not fully represent habitual steady‐state, shod walking in daily life. Because SIS‐rearfoot reflects a composite clinical construct and may be more variable than imaging‐based measures, rearfoot findings should be considered complementary and interpreted cautiously. Cluster analysis was exploratory and not externally validated. Finally, the RA‐specific shortened Spanish FFI (FFI‐Sp) [45] became available after study initiation; therefore, the earlier validated version was used.

Strengths include a homogeneous remission cohort with metatarsal‐region forefoot pain, objective gait assessment and integrated clinical, radiographic and ultrasound evaluation using graded structural metrics.

## Author Contributions

**Rebeca Bueno Fermoso:** conceptualisation, data curation, formal analysis, investigation, methodology, validation, visualisation, writing – original draft, writing – review and editing. **Rosario Morales Lozano:** conceptualisation, investigation, methodology, supervision, validation, writing – review and editing. **Carmen Martínez Rincón:** investigation, methodology, project administration, resources, supervision, writing – review and editing. **Pablo García Fernández:** data curation, formal analysis, investigation, project administration, resources, supervision, visualisation, writing – review and editing. **Juan Miguel López González:** investigation, methodology, project administration, resources, validation, visualisation, writing – review and editing. **María Luz González Fernández:** conceptualisation, investigation, methodology, resources, supervision, validation, writing – review and editing.

## Funding

The authors have nothing to report.

## Ethics Statement

The study was conducted in accordance with the Declaration of Helsinki and approved by the Clinical Research Ethics Committee of Hospital Clínico San Carlos, Madrid, on 21 December 2021 (Ref: 21/719‐E). This research is part of a doctoral thesis.

## Consent

Informed consent was obtained from all subjects involved in the study.

## Conflicts of Interest

The authors declare no conflicts of interest.

## Supporting information


Supporting Information S1

## Data Availability

The datasets generated and analysed during the current study are not publicly available due to patient confidentiality and institutional data protection policies but are available from the corresponding author on reasonable request.
