# Supplementary material for: Clinical and Structural Associations of Disability and Gait Performance in Patients With Rheumatoid Arthritis in Remission and Metatarsal Pain
Source: J Foot Ankle Res. 2026 Feb 12;19(1):e70133. doi: 10.1002/jfa2.70133 (PMC12901661; doi:10.1002/jfa2.70133)
Supplement: Supplementary file 1 — Supporting Information S1 [file JFA2-19-e70133-s001.docx]

**Supplementary material:**

Tabla S1. Spearman Correlation Coefficients Between Clinical, Biomechanical, Ultrasound, Radiographic, and Functional Measures

|  | 1stMTP | TB | HAV-grade | DIS-grade | ER | JSN | SENS | SIS_R | SIS_F | Age | RA years | VAS | DS% | FFI_AL | FFI_D | GV |
| --- | --- | --- | --- | --- | --- | --- | --- | --- | --- | --- | --- | --- | --- | --- | --- | --- |
| 1stMTP | 1.000 | -0.040 | 0.374** | 0.359** | -0.002 | 0.378** | 0.358** | 0.349** | 0.345** | 0.456** | 0.424** | 0.227* | 0.402** | 0.437** | 0.437** | -0.543** |
| TB | -0.040 | 1.000 | -0.062 | -0.150 | -0.151 | -0.177 | -0.074 | 0.183 | -0.385** | -0.040 | -0.127 | 0.179 | 0.231* | 0.129 | 0.136 | -0.041 |
| HAV-grade | 0.374** | -0.062 | 1.000 | 0.513** | 0.049 | 0.303** | 0.354** | 0.304** | 0.633** | 0.625** | 0.266* | 0.047 | 0.227 | 0.288** | 0.290** | -0.417** |
| DIS-grade | 0.359** | -0.150 | 0.513** | 1.000 | 0.457** | 0.744** | 0.842** | 0.386** | 0.739** | 0.613** | 0.558** | 0.052 | 0.206 | 0.229* | 0.234* | -0.491** |
| ER | -0.002 | -0.151 | 0.049 | 0.457** | 1.000 | 0.427** | 0.595** | 0.129 | 0.218 | 0.257* | 0.260* | -0.082 | 0.185 | -0.156 | -0.151 | -0.121 |
| JSN | 0.378** | -0.177 | 0.303** | 0.744** | 0.427** | 1.000 | 0.888** | 0.373** | 0.593** | 0.450** | 0.505** | 0.189 | 0.181 | 0.260* | 0.268* | -0.467** |
| SENS | 0.358** | -0.074 | 0.354** | 0.842** | 0.595** | 0.888** | 1.000 | 0.386** | 0.637** | 0.475** | 0.559** | 0.154 | 0.255* | 0.192 | 0.199 | -0.525** |
| SIS_R | 0.349** | 0.183 | 0.304** | 0.386** | 0.129 | 0.373** | 0.386** | 1.000 | 0.331** | 0.240* | 0.296** | 0.297** | 0.175 | 0.405** | 0.415** | -0.224* |
| SIS_F | 0.345** | -0.385** | 0.633** | 0.739** | 0.218 | 0.593** | 0.637** | 0.331** | 1.000 | 0.496** | 0.521** | -0.040 | 0.204 | 0.146 | 0.151 | -0.387** |
| Age | 0.456** | -0.040 | 0.625** | 0.613** | 0.257* | 0.450** | 0.475** | 0.240* | 0.496** | 1.000 | 0.439** | 0.033 | 0.280* | 0.199 | 0.196 | -0.551** |
| RA years | 0.424** | -0.127 | 0.266* | 0.558** | 0.260* | 0.505** | 0.559** | 0.296** | 0.521** | 0.439** | 1.000 | 0.199 | 0.193 | 0.221* | 0.218 | -0.353** |
| VAS | 0.227* | 0.179 | 0.047 | 0.052 | -0.082 | 0.189 | 0.154 | 0.297** | -0.040 | 0.033 | 0.199 | 1.000 | 0.148 | 0.388** | 0.392** | -0.233* |
| DS% | 0.402** | 0.231* | 0.227 | 0.206 | 0.185 | 0.181 | 0.255* | 0.175 | 0.204 | 0.280* | 0.193 | 0.148 | 1.000 | 0.101 | 0.116 | -0.260* |
| FFI_AL | 0.437** | 0.129 | 0.288** | 0.229* | -0.156 | 0.260* | 0.192 | 0.405** | 0.146 | 0.199 | 0.221* | 0.388** | 0.101 | 1.000 | 0.999** | -0.369** |
| FFI_D | 0.437** | 0.136 | 0.290** | 0.234* | -0.151 | 0.268* | 0.199 | 0.415** | 0.151 | 0.196 | 0.218 | 0.392** | 0.116 | 0.999** | 1.000 | -0.372** |
| GV | -0.543** | -0.041 | -0.417** | -0.491** | -0.121 | -0.467** | -0.525** | -0.224* | -0.387** | -0.551** | -0.353** | -0.233* | -0.260* | -0.369** | -0.372** | 1.000 |

**Note:** *p* < 0.05; **p** < 0.01 (two-tailed Spearman correlation).

**Abbreviations:** 1stMTP, first metatarsophalangeal joint stiffness score; TB, tailor’s bunion; HAV-grade, hallux valgus grade; DIS-grade, summed metatarsophalangeal dislocation grade (0–30); ER, erosions; JSN, joint space narrowing; SENS, Simple Erosion Narrowing Score; SIS_R (SIS-rearfoot), Structural Index Score – rearfoot component; SIS_F (SIS-forefoot), Structural Index Score – forefoot component; Age, age (years); RA years, rheumatoid arthritis duration (years); VAS, visual analogue scale; DS%, double-support time; FFI_AL, Foot Function Index – Activity Limitation subscale; FFI_D, Foot Function Index – Disability subscale; GV, gait velocity.

**Multivariable análisis**

Age- and BMI-adjusted models adding each clinical, structural and inflammatory variable individually are reported in tables S2–S3 and were used to explore domain-specific associations.

For each outcome (FFI-D, FFI-AL, gait velocity and double-support time), we fitted linear regression models adjusted for age and BMI. Each candidate predictor was then entered **separately**, one at a time, into the age + BMI base model to estimate age- and BMI-adjusted associations and to aid interpretation across predictor domains. For each model, we report the unstandardised coefficient (B), standard error (SE), standardised β, p-value, and ΔR² (F-change p-value).

Table S2. Individual linear regression models (Age + BMI base model) for FFI-Disability and FFI-Activity Limitation

| Predictor | B | SE | β | p | 95% CI (B) | ΔR² (p-change) | Total R² | Base modification (Age / BMI) |
| --- | --- | --- | --- | --- | --- | --- | --- | --- |
| FFI-D |  |  |  |  |  |  |  |  |
| Age (base) | 0.81 | 0.28 | 0.32 | 0.004 | 0.27 to 1.36 | — | 0.101 | — |
| BMI (base) | 0.19 | 0.78 | 0.03 | 0.808 | −1.36 to 1.74 | — |  | — |
| Added predictor |  |  |  |  |  |  |  |  |
| VAS | 5.63 | 1.46 | 0.38 | <0.001 | 2.73 to 8.54 | 0.146 (<0.001) | 0.247 | 0.002/ 0.571 |
| GS | −2.72 | 0.90 | −0.33 | 0.003 | −4.52 to −0.92 | 0.095 (0.003) | 0.196 | 0.046 / 0.592 |
| PD | 1.54 | 5.43 | 0.03 | 0.777 | −9.28 to 12.36 | 0.001 (0.777) | 0.102 | 0.004 / 0.840 |
| SENS | 0.76 | 0.51 | 0.17 | 0.144 | −0.26 to 1.78 | 0.025 (0.144) | 0.126 | 0.040 / 0.898 |
| JSN | 1.43 | 0.81 | 0.21 | 0.079 | −0.17 to 3.04 | 0.035 (0.079) | 0.136 | 0.049 / 0.850 |
| ER | −2.50 | 0.87 | −0.31 | 0.005 | −4.22 to −0.77 | 0.087 (0.005) | 0.188 | <0.001 / 0.699 |
| SIS-forefoot | 0.78 | 0.49 | 0.19 | 0.113 | −0.19 to 1.76 | 0.029 (0.113) | 0.130 | 0.033 / 0.527 |
| SIS-rearfoot | 3.90 | 1.39 | 0.31 | 0.006 | 1.14 to 6.66 | 0.084 (0.006) | 0.185 | 0.042 / 0.610 |
| DIS | −0.128 | 0.789 | −0.019 | 0.872 | −1.701 to 1.445 | 0.000 (0.872) | 0.088 | 0.016 / 0.656 |
| DIS-grade | 0.09 | 0.04 | 0.34 | 0.011 | 0.01 to 0.17 | 0.074 (0.011) | 0.175 | 0.344 / 0.957 |
| HAV | 1.843 | 3.642 | 0.059 | 0.614 | −5.410 to 9.096 | 0.003 (0.614) | 0.104 | 0.013 / 0.751 |
| HAV-grade | 4.42 | 1.81 | 0.33 | 0.017 | 0.82 to 8.02 | 0.065 (0.017) | 0.166 | 0.437 / 0.868 |
| Digital deformities | −0.077 | 1.153 | −0.008 | 0.947 | −2.374 to 2.219 | 0.000 (0.947) | 0.090 | 0.012 / 0.823 |
| TB | 6.95 | 3.67 | 0.23 | 0.062 | −0.36 to 14.26 | 0.040 (0.062) | 0.141 | 0.003 / 0.457 |
| 1st MTP (0–4) | 10.19 | 2.36 | 0.51 | <0.001 | 6.71 to 16.13 | 0.209 (<0.001) | 0.310 | 0.346 / 0.856 |
| FFI-AL |  |  |  |  |  |  |  |  |
| Age (base) | 0.75 | 0.26 | 0.31 | 0.005 | 0.23 to 1.27 | — | 0.096 | — |
| BMI (base) | −0.03 | 0.73 | −0.00 | 0.970 | −1.49 to 1.44 | — |  | — |
| Added predictor |  |  |  |  |  |  |  |  |
| VAS | 5.18 | 1.38 | 0.37 | <0.001 | 2.47 to 7.90 | 0.139 (<0.001) | 0.236 | 0.003 / 0.174 |
| GS | −2.29 | 0.86 | −0.29 | 0.010 | −3.99 to −0.60 | 0.076 (0.010) | 0.172 | 0.046 / 0.150 |
| PD | 1.93 | 5.13 | 0.04 | 0.708 | −8.12 to 11.98 | 0.002 (0.708) | 0.098 | 0.005 / 0.930 |
| JSN | 1.27 | 0.76 | 0.20 | 0.099 | −0.22 to 2.77 | 0.032 (0.099) | 0.128 | 0.052 / 0.927 |
| SIS-forefoot | 0.65 | 0.46 | 0.16 | 0.164 | −0.26 to 1.56 | 0.023 (0.164) | 0.120 | 0.033 / 0.833 |
| SIS-rearfoot | 3.30 | 1.32 | 0.28 | 0.015 | 0.71 to 5.89 | 0.068 (0.015) | 0.164 | 0.042 / 0.480 |
| DIS | −0.284 | 0.767 | −0.044 | 0.712 | −1.813 to 1.245 | 0.002 (0.712) | 0.088 | 0.016 / 0.489 |
| DIS-grade | 0.596 | 0.365 | 0.216 | 0.107 | −0.132 to 1.323 | 0.030 (0.107) | 0.127 | 0.175 / 0.887 |
| HAV | 1.354 | 3.442 | 0.046 | 0.695 | −5.500 to 8.208 | 0.002 (0.695) | 0.098 | 0.016 / 0.981 |
| HAV-grade | 3.67 | 1.72 | 0.29 | 0.037 | 0.29 to 7.04 | 0.050 (0.037) | 0.147 | 0.367 / 0.910 |
| Digital deformities | −0.182 | 1.088 | −0.020 | 0.868 | −2.350 to 1.985 | 0.000 (0.868) | 0.085 | 0.013 / 0.955 |
| TB | 5.84 | 3.48 | 0.21 | 0.097 | −0.98 to 12.67 | 0.032 (0.097) | 0.128 | 0.004 / 0.379 |
| 1st MTP | 10.19 | 2.27 | 0.48 | <0.001 | 5.75 to 14.64 | 0.188 (<0.001) | 0.284 | 0.333 / 0.897 |

**Abbreviations:** VAS, visual analogue scale; BMI, body mass index; RA years, disease duration in years; FFI-D, Foot Function Index – Disability subscale; FFI-AL, Foot Function Index – Activity Limitation subscale; V, gait velocity (m/s); DS, double-support time (%); GS, grey-scale synovitis; PD, power Doppler; SENS, Simple Erosion Narrowing Score; JSN, joint space narrowing; ER, erosions; SIS-forefoot, Structural Index Score – forefoot component; SIS-rearfoot, Structural Index Score – midfoot and rearfoot component; DIS, simple dislocation count (1st–5th MTP, yes/no); DIS-total / DIS-grade, summed dislocation grade (1st–5th MTP); HAV, presence of hallux valgus; HAV-grade, hallux valgus grade (Manchester scale, bilateral); 1st MTP, first metatarsophalangeal joint stiffness score (bilateral); TB, tailor’s bunion (5th MTP).

Base modification shows the p-values for age and BMI after adding the predictor.

Table S3**.** Linear regression models for gait performance parameters (gait velocity and double support)

| Predictor | B | SE | β | p | 95% CI (B) | ΔR² (p-change) | Total R² | Base modification (Age / BMI) |
| --- | --- | --- | --- | --- | --- | --- | --- | --- |
| GV, m/s |  |  |  |  |  |  |  |  |
| Age  (base) | −0.02 | 0.00 | −0.56 | <0.001 | −0.02 to −0.01 | — | 0.312 |  |
| BMI  (base) | −0.01 | 0.01 | −0.07 | 0.450 | −0.02 to 0.01 | — |  |  |
| Added predictor |  |  |  |  |  |  |  |  |
| VAS | −0.02 | 0.02 | −0.12 | 0.193 | −0.05 to 0.01 | 0.015 (0.193) | 0.327 | <0.001 / 0.388 |
| GS | 0.03 | 0.01 | 0.26 | 0.010 | 0.01 to 0.04 | 0.058 (0.010) | 0.370 | <0.001 / 0.308 |
| PD | −0.05 | 0.06 | −0.09 | 0.369 | −0.16 to 0.06 | 0.007 (0.369) | 0.319 | <0.001 / 0.531 |
| SENS | −0.02 | 0.01 | −0.35 | <0.001 | −0.03 to −0.01 | 0.102 (<0.001) | 0.414 | <0.001 / 0.597 |
| JSN | −0.02 | 0.01 | −0.30 | 0.003 | −0.04 to −0.01 | 0.075 (0.003) | 0.386 | <0.001 / 0.482 |
| SIS-forefoot | −0.01 | 0.01 | −0.18 | 0.089 | −0.02 to 0.00 | 0.026 (0.089) | 0.338 | <0.001 / 0.293 |
| SIS-rearfoot | −0.01 | 0.02 | −0.04 | 0.672 | −0.04 to 0.02 | 0.002 (0.672) | 0.314 | <0.001 / 0.544 |
| DIS | −0.006 | 0.009 | −0.075 | 0.475 | −0.024 to 0.011 | 0.005 (0.475) | 0.306 | <0.001 / 0.708 |
| DIS-grade | −0.011 | 0.004 | −0.333 | 0.004 | −0.019 to −0.004 | 0.072 (0.004) | 0.384 | 0.002 / 0.547 |
| HAV | 0.001 | 0.038 | 0.002 | 0.985 | −0.074 to 0.076 | 0.000 (0.985) | 0.312 | <0.001 / 0.461 |
| HAV-grade | −0.03 | 0.02 | −0.19 | 0.125 | −0.07 to 0.01 | 0.021 (0.125) | 0.333 | <0.001 / 0.479 |
| Digital deformities | 0.001 | 0.012 | 0.010 | 0.926 | −0.022 to 0.024 | 0.000 (0.926) | 0.275 | <0.001 / 0.436 |
| 1st MTP | −0.10 | 0.03 | −0.38 | <0.001 | −0.15 to −0.05 | 0.113 (<0.001) | 0.425 | <0.001 / 0.463 |
| TB | −0.01 | 0.04 | −0.01 | 0.897 | −0.08 to 0.07 | 0.000 (0.897) | 0.312 | <0.001 / 0.563 |
| DS, (% gait cycle) |  |  |  |  |  |  |  |  |
| Age (base) | 0.085 | 0.087 | 0.111 | 0.332 | −0.088 to 0.257 | — | 0.061 |  |
| BMI (base) | 0.443 | 0.228 | 0.220 | 0.056 | −0.012 to 0.898 | — |  |  |
| Added predictor |  |  |  |  |  |  |  |  |
| VAS | 1.105 | 0.461 | 0.266 | 0.019 | 0.186 to 2.024 | 0.069 (0.019) | 0.130 | 0.221 / 0.029 |
| GS | −0.490 | 0.280 | −0.208 | 0.085 | −1.049 to 0.069 | 0.038 (0.085) | 0.099 | 0.726 / 0.045 |
| PD | −2.441 | 1.535 | −0.181 | 0.116 | −5.501 to 0.619 | 0.032 (0.116) | 0.093 | 0.427 / 0.034 |
| JSN | 0.065 | 0.238 | 0.034 | 0.784 | −0.409 to 0.539 | 0.001 (0.784) | 0.062 | 0.425 / 0.058 |
| SIS-forefoot | 0.128 | 0.144 | 0.112 | 0.378 | −0.159 to 0.415 | 0.010 (0.378) | 0.071 | 0.596 / 0.043 |
| SIS-rearfoot | 0.305 | 0.422 | 0.088 | 0.472 | −0.536 to 1.145 | 0.007 (0.472) | 0.068 | 0.463 / 0.097 |
| DIS | 0.117 | 0.230 | 0.066 | 0.613 | −0.342 to 0.575 | 0.004 (0.613) | 0.041 | 0.960 / 0.107 |
| DIS-grade | 0.207 | 0.115 | 0.255 | 0.077 | −0.023 to 0.436 | 0.040 (0.077) | 0.101 | 0.743 / 0.055 |
| HAV | 1.696 | 1.062 | 0.195 | 0.115 | −0.421 to 3.814 | 0.032 (0.115) | 0.093 | 0.746 / 0.033 |
| HAV-grade | 1.401 | 0.551 | 0.367 | 0.013 | 0.302 to 2.499 | 0.077 (0.013) | 0.138 | 0.379 / 0.083 |
| Digital deformities | 0.281 | 0.327 | 0.106 | 0.393 | −0.372 to 0.935 | 0.010 (0.393) | 0.074 | 0.557 / 0.049 |
| 1st MTP | 2.432 | 0.746 | 0.382 | 0.002 | 0.944 to 3.919 | 0.121 (0.002) | 0.181 | 0.684 / 0.036 |
| TB | 3.332 | 1.067 | 0.402 | 0.003 | 1.204 to 5.460 | 0.112 (0.003) | 0.173 | 0.143 / 0.983 |

Abbreviations: VAS, visual analogue scale; BMI, body mass index; RA years, disease duration in years; FFI-D, Foot Function Index – Disability subscale; FFI-AL, Foot Function Index – Activity Limitation subscale; V, gait velocity (m/s); DS, double-support time (%); GS, grey-scale synovitis; PD, power Doppler; SENS, Simple Erosion Narrowing Score; JSN, joint space narrowing; ER, erosions; SIS-forefoot, Structural Index Score – forefoot component; SIS-rearfoot, Structural Index Score – midfoot and rearfoot component; DIS, simple dislocation count (1st–5th MTP, yes/no); DIS-total / DIS-grade, summed dislocation grade (1st–5th MTP); HAV, presence of hallux valgus; HAV-grade, hallux valgus grade (Manchester scale, bilateral); 1st MTP, first metatarsophalangeal joint stiffness score (bilateral); TB, tailor’s bunion (5th MTP).

Base modification shows the p-values for age and BMI after adding the predictor.

**Sensibity multivariable parsimonious models**

Linear regression models. Base: Age+BMI+VAS+GS+SIS-rearfoot+Added predictor.

**Tables S4.S5.S6.S7.**

Table S4.. Parsimonious model. FFI-D.

| Predictor | B | SE | β | p | ΔR² (p-change) | Total R² | Base modification (Age; BMI; VAS; GS; SIS rearfoot) |
| --- | --- | --- | --- | --- | --- | --- | --- |
| Age (base) | 0,417 | 0,264 | 0,163 | 0,118 | — | 0,353 | — |
| BMI (base) | 0,110 | 0,707 | 0,015 | 0,877 | — |  | — |
| VAS (base) | 4,265 | 1,460 | 0,290 | 0,005 | — |  | — |
| GS (synovitis) (base) | -2,458 | 0,826 | -0,294 | 0,004 | — |  | — |
| SIS rearfoot (base) | 2,614 | 1,320 | 0,211 | 0,051 | — |  | — |
| Added predictor |  |  |  |  |  |  |  |
| SIS forefoot | 0,733 | 0,466 | 0,175 | 0,120 | 0,021 (0,120) | 0,374 | Age X/X; BMI X/X; VAS V/V; GS V/V; SIS rearfoot X/X |
| SENS | 0,488 | 0,473 | 0,112 | 0,305 | 0,009 (0,305) | 0,362 | Age X/X; BMI X/X; VAS V/V; GS V/V; SIS rearfoot X/X |
| JSN | 0,868 | 0,744 | 0,125 | 0,247 | 0,012 (0,247) | 0,365 | Age X/X; BMI X/X; VAS V/V; GS V/V; SIS rearfoot X/X |
| ER | -1,986 | 0,794 | -0,243 | 0,015 | 0,050 (0,015) | 0,403 | Age X/V; BMI X/X; VAS V/V; GS V/V; SIS rearfoot X/V |
| DIS-grade | 0,594 | 0,346 | 0,203 | 0,091 | 0,025 (0,091) | 0,378 | Age X/X; BMI X/X; VAS V/V; GS V/V; SIS rearfoot X/X |
| HAV-grade | 3,199 | 1,627 | 0,239 | 0,053 | 0,032 (0,053) | 0,385 | Age X/X; BMI X/X; VAS V/V; GS V/V; SIS rearfoot X/X |
| 1st MTP | 7,471 | 2,570 | 0,331 | 0,005 | 0,066 (0,005) | 0,419 | Age X/X; BMI X/X; VAS V/V; GS V/X; SIS rearfoot X/X |
| HAV-grade  + 1st MTP |  |  |  |  |  |  |  |
| HAV-grade | 2,764 | 1,567 | 0,207 | 0,082 | 0,090 (0,004) | 0,443 | Age X/X; BMI X/X; VAS V/V; GS V/X; SIS rearfoot X/X |
| 1st MTP | 7,018 | 2,547 | 0,311 | 0,007 |  |  |  |

Table S5. Parsimonious model. FFI-AL.

| Predictor | B | SE | β | p | ΔR² (p-change) | Total R² | Base modification  (Age; BMI; VAS; GS; SIS rearfoot) |
| --- | --- | --- | --- | --- | --- | --- | --- |
| Age (base) | 0,427 | 0,255 | 0,177 | 0,098 | — | 0,316 | — |
| BMI (base) | -0,016 | 0,685 | -0,002 | 0,982 | — |  | — |
| VAS (base) | 4,100 | 1,414 | 0,296 | 0,005 | — |  | — |
| GS (base) | -2,043 | 0,800 | -0,259 | 0,013 | — |  | — |
| SIS-rearfoot (base) | 2,038 | 1,278 | 0,174 | 0,115 | — |  | — |
| Added predictor |  |  |  |  |  |  |  |
| SIS forefoot | 0,651 | 0,452 | 0,165 | 0,154 | 0,019 (0,154) | 0,335 | Age X/X; BMI X/X; VAS V/V; GS V/V; SIS rearfoot X/X |
| SENS | 0,326 | 0,459 | 0,079 | 0,480 | 0,005 (0,480) | 0,321 | Age X/X; BMI X/X; VAS V/V; GS V/V; SIS rearfoot X/X |
| JSN | 0,787 | 0,722 | 0,120 | 0,279 | 0,011 (0,279) | 0,327 | Age X/X; BMI X/X; VAS V/V; GS V/V; SIS rearfoot X/X |
| ER | -1,999 | 0,766 | -0,259 | 0,011 | 0,058 (0,011) | 0,374 | Age X/V; BMI X/X; VAS V/V; GS V/X; SIS rearfoot X/X |
| DIS-grade | 0,445 | 0,338 | 0,162 | 0,192 | 0,016 (0,192) | 0,332 | Age X/X; BMI X/X; VAS V/V; GS V/V; SIS rearfoot X/X |
| HAV-grade | 2,649 | 1,587 | 0,211 | 0,099 | 0,025 (0,099) | 0,341 | Age X/X; BMI X/X; VAS V/V; GS V/V; SIS rearfoot X/X |
| 1st MTP | 6,941 | 2,500 | 0,327 | 0,007 | 0,065 (0,007) | 0,381 | Age X/X; BMI X/X; VAS V/V; GS V/X; SIS rearfoot X/X |
| HAV-grade  + 1st MTP |  |  |  |  |  |  |  |
| HAV-grade | 2,242 | 1,534 | 0,178 | 0,148 | 0,082 (0,004) | 0,398 | Age X/X; BMI X/X; VAS V/V; GS V/X; SIS rearfoot X/X |
| 1st MTP | 6,574 | 2,494 | 0,310 | 0,010 |  |  |  |

Table S6. Parsimonious model. GV.

| Predictor | B | SE | β | p | ΔR² (p-change) | Total R² | Base modification (Age; BMI; VAS; GS; SIS rearfoot) |
| --- | --- | --- | --- | --- | --- | --- | --- |
| Age (base) | -0,014 | 0,003 | -0,461 | <0,001 | — | 0,373 | — |
| BMI (base) | -0,007 | 0,008 | -0,078 | 0,417 | — |  | — |
| VAS (base) | -0,013 | 0,017 | -0,076 | 0,441 | — |  | — |
| GS (base) | 0,025 | 0,009 | 0,257 | 0,010 | — |  | — |
| SIS rearfoot (base) | -0,005 | 0,015 | -0,031 | 0,766 | — |  | — |
| Added predictor |  |  |  |  |  |  |  |
| SIS forefoot | -0,011 | 0,005 | -0,223 | 0,042 | 0,035 (0,042) | 0,408 | Age V/V; BMI X/X; VAS X/X; GS V/V; SIS rearfoot X/X |
| SENS | -0,021 | 0,005 | -0,404 | <0,001 | 0,120 (<0,001) | 0,493 | Age V/V; BMI X/X; VAS X/X; GS V/V; SIS rearfoot X/X |
| JSN | -0,026 | 0,008 | -0,318 | 0,002 | 0,076 (0,002) | 0,449 | Age V/V; BMI X/X; VAS X/X; GS V/V; SIS rearfoot X/X |
| ER | -0,006 | 0,009 | -0,060 | 0,546 | 0,003 (0,546) | 0,376 | Age V/V; BMI X/X; VAS X/X; GS V/V; SIS rearfoot X/X |
| DIS-grade | -0,024 | 0,019 | -0,155 | 0,205 | 0,014 (0,205) | 0,387 | Age V/V; BMI X/X; VAS X/X; GS V/V; SIS rearfoot X/X |
| HAV-grade | -0,086 | 0,029 | -0,326 | 0,005 | 0,064 (0,005) | 0,437 | Age V/V; BMI X/X; VAS X/X; GS V/X; SIS rearfoot X/X |
| HAV-grade + 1st MTP |  |  |  |  |  |  |  |
| HAV-grade | -0,019 | 0,018 | -0,123 | 0,298 | 0,073 (0,001) | 0,446 | Age V/V; BMI X/X; VAS X/X; GS V/X; SIS rearfoot X/X |
| 1st MTP | -0,082 | 0,030 | -0,314 | 0,007 |  |  |  |

Table S7. Parsimonious model. DS(%).

| Predictor | B | SE | β | p | ΔR² (p-change) | Total R² | Base modification (Age; BMI; VAS; GS; SIS rearfoot) |
| --- | --- | --- | --- | --- | --- | --- | --- |
| Age (base) | 0,086 | 0,090 | 0,114 | 0,344 | — | 0,173 | — |
| BMI (base) | 0,532 | 0,228 | 0,263 | 0,022 | — |  | — |
| VAS (base) | 1,094 | 0,480 | 0,265 | 0,026 | — |  | — |
| GS (base) | -0,333 | 0,268 | -0,141 | 0,218 | — |  | — |
| SIS-rearfoot (base) | 0,022 | 0,427 | 0,007 | 0,958 | — |  | — |
| Added predictor |  |  |  |  |  |  |  |
| SIS forefoot | 0,162 | 0,150 | 0,139 | 0,283 | 0,013 (0,283) | 0,186 | Age X/X; BMI V/V; VAS V/V; GS X/X; SIS rearfoot X/X |
| SENS | 0,280 | 0,152 | 0,231 | 0,070 | 0,037 (0,070) | 0,210 | Age X/X; BMI V/V; VAS V/V; GS X/X; SIS rearfoot X/X |
| JSN | 0,015 | 0,241 | 0,008 | 0,951 | 0,000 (0,951) | 0,173 | Age X/X; BMI V/V; VAS V/V; GS X/X; SIS rearfoot X/X |
| ER | -0,097 | 0,277 | -0,042 | 0,726 | 0,001 (0,726) | 0,174 | Age X/X; BMI V/V; VAS V/V; GS X/X; SIS rearfoot X/X |
| DIS-grade | 0,198 | 0,115 | 0,244 | 0,090 | 0,033 (0,090) | 0,206 | Age X/X; BMI V/V; VAS V/V; GS X/X; SIS rearfoot X/X |
| HAV-grade | 1,017 | 0,520 | 0,265 | 0,055 | 0,042 (0,055) | 0,215 | Age X/X; BMI V/V; VAS V/V; GS X/X; SIS rearfoot X/X |
| 1st MTP | 2,239 | 0,843 | 0,360 | 0,010 | 0,074 (0,010) | 0,247 | Age X/X; BMI V/V; VAS V/X; GS X/X; SIS rearfoot X/X |
| HAV-grade + 1st MTP |  |  |  |  |  |  |  |
| HAV-grade | 0,860 | 0,507 | 0,224 | 0,094 | 0,104 (0,004) | 0,277 | Age X/X; BMI V/V; VAS V/V; GS X/X; SIS rearfoot X/X |
| 1st MTP | 2,059 | 0,839 | 0,331 | 0,017 |  |  |  |

Abbreviations: FFI-D, Foot Function Index–Disability (0–100); FFI-AL, Foot Function Index–Activity Limitation (0–100); GV, gait velocity (m/s); DS, double-support time (% gait cycle); Age, age (years); BMI, body mass index (kg/m²); VAS, visual analogue scale for pain (0–10); GS, grey-scale synovitis; SIS-rearfoot, Structural Index Score–rearfoot; SIS-forefoot, Structural Index Score–forefoot; JSN, joint space narrowing; ER, erosions; SENS, radiographic structural score (as defined in your methods); HAV-grade, hallux valgus grade (Manchester scale, bilateral sum); 1st MTP, first metatarsophalangeal joint stiffness/limitation score. B, unstandardized coefficient; SE, standard error; β, standardized coefficient; ΔR², change in R² vs base model; p-change, p-value for ΔR².

Simbols: Base modification:

The notation is shown as base model / modified model. X indicates that the covariate is kept unchanged, whereas V indicates that the covariate is modified (removed or substituted) in that model specification.

- X/X = kept in the base model and kept in the modified model.
- X/V = kept in the base model but modified (removed/substituted) in the modified model.
- V/X = modified in the base model but kept in the modified model.
- V/V = modified in both the base and the modified model.
